# A Naturally Occurring Antioxidant Complex from Unripe Grapes: The Case of Sangiovese (v. *Vitis* *vinifera*)

**DOI:** 10.3390/antiox7020027

**Published:** 2018-02-08

**Authors:** Giovanna Fia, Claudio Gori, Ginevra Bucalossi, Francesca Borghini, Bruno Zanoni

**Affiliations:** 1 Dipartimento di Gestione dei Sistemi Agrari, Alimentari e Forestali, University of Florence, Via Donizetti, 6, 50144 Firenze, Italy; ginevra.bucalossi@unifi.it (G.B.); bruno.zanoni@unifi.it (B.Z.); 2 Vino Vigna, Via Claudio Monteverdi, 9, 50053 Empoli, Italy; c.gori@vinovigna.com; 3 ISVEA Srl, Servizi Analitici di Eccellenza per i Settori Enologico, Viticolo e il Comparto Alimentare, Via Basilicata 1/3, Poggibonsi, 53036 Siena, Italy; f.borghini@isvea.it

**Keywords:** unripe grapes, Sangiovese, phenolic compounds, antioxidant activity, solid-liquid extraction

## Abstract

The wine industry is well known for its production of a large amount of wastes and by-products. Among them, unripe grapes from thinning operations are an undervalued by-product. Grapes are an interesting source of natural antioxidants such as flavonoids, non-flavonoids and stilbenes. A potential strategy to exploit unripe grapes was investigated in this study. Juice from unripe grapes, v. Sangiovese, was obtained by an innovative technique of solid-liquid extraction without the use of solvents. The juice was dried by a spray-drying technique with the addition of arabic gum as support to obtain powder; juice and powder were characterized for antioxidant activity, phenolic concentration and profile. Phenolic acids, flavonols, flava-3-ols, procyanidins and resveratrol were detected in the juice and powder. The powder was used as anti-browning additive in white wine to test the potential re-use of the unripe grapes in the wine industry. The results indicated that the antioxidant complex from unripe grapes contributed to increasing the anti-browning capacity of white wine. Other applications, such as food and nutraceutical products development, can be considered for the antioxidant complex extracted from unripe grapes. In conclusion, the method proposed in this study may contribute to the exploitation of unripe grapes as a by-product of the winemaking process.

## 1. Introduction

Grapes are rich in bio-active compounds such as vitamins and polyphenols, which act as powerful antioxidants able to scavenge diverse reactive oxygen species (ROS) or inhibit their formation, and hence the oxidation of biomolecules [1]. Polyphenols are involved in the transfer of electrons to free radicals, chalation metal catalyst (Fe^2+^ and Cu^+^), reduction of alpha-tocopherol radicals, and inhibition of oxidase [2]. Beyond the usual antioxidant activities, the protective effects of polyphenols could be due to their ability to act as modulators of cell signaling [3,4,5]. The antioxidant properties of grapes have been associated with their phenolic composition and, in particular, with the high content in anthocyanins, flavonols, flava-3-ols, procyanidins and phenolic acids [6,7]. Other polyphenols, such as resveratrol, unique to red grapes, can contribute to the health properties of matrices derived from this fruit. Polyphenols in grapes exist in a free form but the majority of these compounds are glycosides of different sugar units and acylated at different positions of the polyphenol skeleton. Each class of grape phenol compounds showed different antioxidant capacity in vitro and in vivo, with often inconsistent data [6,8]. The differences observed among in vitro and in vitro tests could be ascribed to the bioavailability of polyphenols that contribute to the effectiveness of these compounds in a biological system [9].

Winemaking is well-known for its generation of large amounts of organic wastes and by-products, such as marcs, pomace and lees. The industrial wine sector is exploring solutions to develop marketable products resulting from the exploitation of the industry’s wastes, by converting waste materials into food ingredients and other bio-products with high added value [10]. One undervalued by-product of the wine sector is unripe grapes derived from thinning operations carried out to increase the quality of production. The aim of thinning is to remove bunches that do not have the ability to achieve a suitable maturation, thus promoting the maturation of those that remain on the plant. Unripe grapes of low quality, such as table grapes, are traditionally processed into various food products [11]. A potential use of unripe grapes in winemaking to reduce the alcohol concentration and pH of wine was investigated by other authors, who observed a partial reduction of alcohol content and simultaneous decrease of pH of both Cabernet Sauvignon and Merlot experimental wines [12]. Although the bioactive compounds and antioxidant activities of grapes at maturity and by-products of the wine production chain have been the subject of many investigations, unripe grapes and their potential application in the food industry have been scarcely studied [13,14]. Unripe grapes are a source of natural antioxidants such as polyphenols and resveratrol. Indeed, the biosynthesis of these compounds can start before veraison (change of color), when berries are still green, and continue over the course of ripening. It is known that the phenolic composition of grapes depends on many factors, such as variety, maturation stage and environmental conditions [15]. Antioxidant compounds from unripe grapes are potentially exploitable in the food industry as functional ingredients and protective agents against oxidation. Sangiovese is one of the most cultivated varieties of grapes in Italy, and has considerable economic importance. The phenolic composition of Sangiovese has been well studied [15], while information about the antioxidant properties of unripe grapes, v. Sangiovese, and their potential applications are lacking.

The aim of the present study was to obtain an antioxidant complex from unripe grapes, v. Sangiovese, and to evaluate its composition and antioxidant capacity. In addition, the potential protective effect toward white wine oxidation was investigated.

## 2. Materials and Methods

### 2.1. Chemicals

Standards, solvents and reagents were purchased from Sigma-Aldrich (Milan, Italy), except for quercetin-3-Oglucoside, quercetin-3-*O*-glucuronide and rutin, which were supplied by HWI Analytik GmbH (Rülzheim, Germany). Methanol and ethanol were supplied by Carlo Erba (Milan, Italy). Ultrapure water was obtained from a Milli-Q Gradient water purification system (Thermo Scientific, Waltham, MA, USA).

### 2.2. Sangiovese Grapes

Sangiovese grapes were obtained from thinning operations during “green harvesting” on a vineyard located in Lucca, Tuscany, Italy. The grapes were manually harvested on 18 August 2015 during berry ripening at growth stage 36 according to the modified Eichhorn-Lorenz (E-L) system [16]. Only the healthy grapes were transported to the winery in small cases for further operations.

### 2.3. Wines

Three unfinished wines, Viognier, Chardonnay and Bellone (sparkling base wine), vintage 2015, were provided by the La Torre farm, Velletri, Italy. These commercial wines were used in this study to evaluate the anti-browning capacity of the anti-oxidant complex from unripe grapes.

### 2.4. Preparation of Juice and Powder

**Figure 1:**
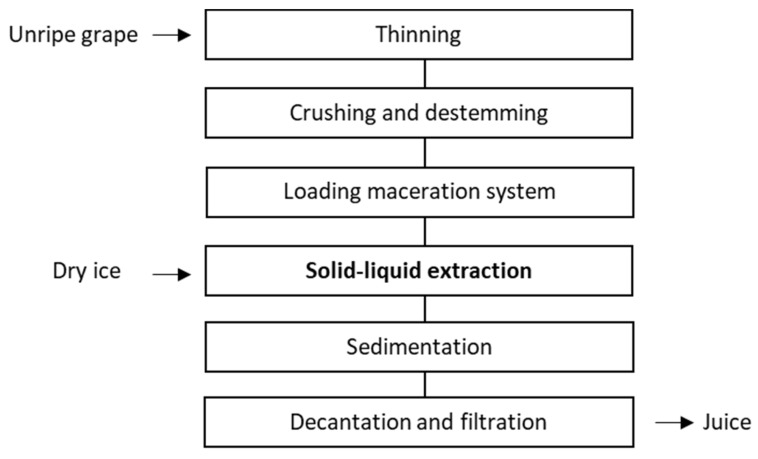
Scheme of process for juice production from unripe grapes.

The grapes were crushed and destemmed at the winery, using a Delta E2 destemmer (Bucher Vaslin, Zurich, Switzerland), then transferred to an industrial system [17] for the solid-liquid extraction phase, also performed at the winery (Figure 1).

The system is a stainless-steel tank, of 12,500 L capacity, covered for 75% of its surface by a low-temperature thermostated jacket. Inside the system, there are four whorls to stir up the crushed grapes. A device automatically controls temperature and remixing. The system is also equipped with accessories for loading the grapes, draining the juice, and the discharge of semi-solid residue (pomace). A total of 650 kg of grapes were processed with the addition of 1 kg of dry ice to every 10 kg of grapes. The grapes were remixed every 6 h for 30 min, for a total period of 3 days, at a temperature of 6 °C. During the process, samples of juice (500 mL) were taken immediately after mixing, at the start (2 h), and after 6, 18, 24, 30, 42, 48, 54, 66 and 72 h. Furthermore, after 2 and 72 h, three samples of juice (500 mL) were taken for phenolic composition evaluation. All samples were stored at −20 °C for further analysis. After 72 h, the product was maintained still inside the system at 6 °C for 48 h for sedimentation. After this period, the juice was decanted and large-particle (Ø 1 mm) filtered before freezing. The juice (250 L) was stored at −20 °C until the moment of spray-drying. After thawing, the juice was combined with arabic gum (16% *w*/*v*) (Nexira Food, Rouen, France) as support for spray-drying, mixed well and spray-dried to obtain powder. It was necessary to use a support because of the sugar content (153 g/L) of the juice. Arabic gum was chosen from among different types of support because its use is allowed in winemaking. Spray-drying was performed using an industrial turbine spry-dryer (Gea Niro, Milan, Italy). The rotational speed of the turbine was 18,000 revolutions per minute, and the flow rate of the peristaltic pump automatically controlled the drying air temperature, which was 180 °C (input) and 80 °C (outlet). The powder, placed in polyethylene pouches, was stored in a desiccator containing silica, in the dark, for further analysis.

### 2.5. General Analyses

Alcohol, reducing sugar, total acidity, pH, total and free sulphur dioxide were evaluated in duplicate according to the official or usual methods recommended by the International Organisation of the Vine and Wine, (OIV) [18].

### 2.6. Total Polyphenols (TP) Determination

Total polyphenols were quantified according to the Folin-Ciocalteau (FC) method [19] with some modifications. Undiluted juice and powder solutions (10%, *w*/*v*) prepared in distilled water were filtered on a membrane (Ø 0.45 µm). Phenolic compounds were purified from 1 mL of undiluted juice or powder solution on C18 Sep-pak cartridge (Waters, Milan, Italy) following the method described by Di Stefano et al. [20]. 4 mL of sodium carbonate (10%, *w*/*v*) was added to 1 mL of each sample, mixed well, and left to stand for 5 min. A volume of 1 mL of diluted FC reagent was added to the mixture, and it was then well shaken. Samples were left in the dark for 90 min at room temperature, and then the absorbance was measured spectrophotometrically at 700 nm with a Perkin Elmer Lambda 10 spectrophotometer (Waltham, MA, USA). TP was expressed as (+)-catechin equivalents (CATeq)/L of juice or (CATeq)/g powder. A standard curve was obtained with (+)-catechin solutions at concentrations ranging from 5 to 500 mg/L.

### 2.7. Total Anthocyanins Determination

Wine sample was diluted with a solution consisting of C_2_H_5_OH/H_2_O/HCl  =  69/30/1 (*v/v/v*) and the absorbance was measured at 540 nm [20], using a Perkin Elmer Lambda 10 spectrophotometer. The total anthocyanin (TA) content was expressed as malvidin-3-glucoside equivalents and calculated using the following formula:

TA_540 nm_ (mg/L) = A_540nm_ × 16.7 × d

where A_540nm_, absorbance at the wavelength of 540 nm; d, dilution; 16.7, molar extinction coefficient of malvidin-3-glucoside.

### 2.8. 2,2-Diphenyl-1-Picryhydrazil (DPPH) Antioxidant Test

Free-radical-scavenging activity was evaluated by the 2,2-diphenyl-1-picryhydrazil (DPPH) test [21], with some modifications. Briefly, a solution of DPPH (6 × 10^−5^ M) was prepared by dissolving 0.236 mg of DPPH in 100 mL of methanol. Undiluted juice or powder solution (10%, *w*/*v*) prepared in distilled water were filtered on a membrane (Ø 0.45 μm). For the reaction, 0.1 mL of either undiluted juice or powder solution was mixed with 3.9 mL of DPPH stock solution. For the reference sample, 0.1 mL of solvent was added to 3.9 mL of DPPH solution to measure the maximum DPPH absorbance. Samples were left in the dark for 30 min at 30 °C for the reaction and immediately afterwards the decrease of absorbance was measured at 515 nm with a Perkin Elmer Lambda 10 spectrophotometer (Waltham, MA, USA). Antioxidant activity was expressed as µmoL of Trolox equivalents antioxidant capacity (TEAC)/L of juice and (TEAC)/g of powder. Trolox standard solutions were prepared in ethanol at concentrations ranging from 10 to 600 µmoL/L. Each assay was performed in triplicate.

### 2.9. Liquid Chromatography-High-Resolution Mass Spectrometry (LC-HRMS) Analysis

Phenolic compounds and glutathione analysis was performed via liquid chromatography-high-resolution mass spectrometry (LC-HRMS), coupled with a diode array detector (DAD) (Ultimate 3000, Dionex, Thermo Fisher Scientific (Waltham, MA, USA)). All samples and standards were handled to minimize light exposure. The samples were filtrated to 0.45 µm and then analyzed, without any other preparation step. The liquid chromatograph was an Accela (Accela 1250, Thermo Fisher Scientific, (Waltham, MA, USA) equipped with a quaternary pump and a thermostated autosampler. A kinetex F5 column (2.1 × 100 mm 1.7 µm; Phenomenex (Torrance, CA, USA)) was used. Autosampler tray temperature was set at 10 °C, and the column at 40 °C. Gradient elution was performed with water/0.05% formic acid/5 mM ammonium formate (solvent A) and methanol/0.05% formic acid/5 mM ammonium formate (solvent B) at a constant flow rate of 400 µL/min; and injection volume was 3 µL. An increasing linear gradient of solvent B was used. Separation was carried out in 45 min under the following conditions: 0 min, 5% B; 13 min, 5% B; 22 min, 35% B; 24 min, 35% B; 27 min, 90%; 32 min 90% B and from 33 min to 45 min, 5% B. An LTQ Orbitrap Exactive mass spectrometer (Thermo Fisher Scientific, (Waltham, MA, USA)) equipped with an electrospray ionization (ESI) source in negative mode was used to acquire mass spectra in a full mass spectrometry (MS) data dependent MS^2^ experiment. Operation parameters were as follows: source voltage, 4 kV; sheath gas, 35 (arbitrary units); auxiliary gas, 10 (arbitrary units); sweep gas, 0 (arbitrary units); capillary temperature, 300 °C, S-lens radio frequency (RF) level, 60 and automatic gain control (AGC) target, 1 × 10^6^ for MS mode and 2 × 10^5^ for MS^2^ mode. Samples were first analyzed in full MS mode with the resolution set at 70,000, whereas the subsequent analyses were performed in dd-MS^2^ mode with the resolution set at 17,500. An isolation width of 2 amu was used, and precursors were fragmented by stepped normalized collision energy of 25, 30 and 35. The maximum injection time was set to 200 ms with one microscan both for MS mode and for MS^n^ mode. The mass range was from *m*/*z* 150 to 1000. Data analyses were performed using TraceFinder™ 4.1 software (Thermo Fisher Scientific (Waltham, MA, USA)). Peak assignment was carried out on the basis of the exact mass of the molecular ions and the cis and trans forms were recognized by comparison of the retention times with the standard sample. Quantitative analysis was performed by TraceFinder™ 4.1 software (Thermo Fisher Scientific (Waltham, MA, USA)) with external standard method, using a linear regression of five standard solutions of a mix of the above reported substances from 0.05 to 1 g L^−1^. For coutaric and fertaric acids, due to the lack of reference materials, corresponding free acids (coumaric and ferulic acids) were used as standard. The samples out of calibration range were conveniently diluted in 12% water/ethanol solution. The recovery and matrix effects were checked for all the samples by standard additions and consecutive dilutions as well. The analysis was carried out in triplicate. All chromatographic runs were collected in the same work session and the resulting relative standard deviation (RSD) was less than 10%.

### 2.10. HPLC-Determination of Anthocyanins

The relative composition of anthocyanins was determined by high-performance liquid chromatography (HPLC) analysis performed with an HPLC 1290 (AGILENT, Santa Clara, CA, USA), using a reverse phase column and ultraviolet-visible (UV-VIS) detection [17].

### 2.11. Polyphenols Oxidative Medium (POM) Test

The wine’s predisposition towards browning was determined by the so-called POM-test [22,23]. A volume of 15 mL of wine was heated at 60 °C for 1 h, then 60 μL of a 3% hydrogen peroxide solution was added to accelerate the oxidation of color. The browning produced was estimated on the basis of the percent increase (% Oxidation (OX)_H2O2_) of the absorbance at 420 nm and calculated by the following formula:

% OX_H2O2_ = [(A_OX_ − A_BOX_)/A_BOX_] × 100

The absorbance was measured using a Perkin Elmer Lambda 10 spectrophotometer before oxidation (A_BOX_) and after oxidation (A_OX_), after cooling the solution at room temperature. The POM-test was performed in triplicate with and without the addition of the antioxidant complex (powder) at a concentration of 2 g/L.

### 2.12. Statistics Analysis

Chemical analyses were performed in duplicate or triplicate and the data are presented as mean ± standard deviation. Analysis of variance (ANOVA) (Least Significant Differences (LSD), 5% level) was performed using Statgraphics plus 3.1 by Statgraphics (The Plains, VA, USA).

## 3. Results and Discussion

### 3.1. Solid-Liquid Extraction and Juice Composition

The antioxidant complex of unripe grapes was obtained according to the process scheme shown in Figure 1. At the end of processing, juice yield was about 40% of the grapes used. The analytical parameters of the juice were compatible with unripe grapes: total acidity (11.09 g/L as tartaric acid), pH 2.97, l-malic acid 3.43 (g/L), sugar content (153 g/L), non-detectable alcohol. Low temperature (6 °C) prevented unwanted fermentation.

Solid-state carbon dioxide (dry ice) is the most-used cryogen to lower temperature in winemaking. The sublimation of dry ice induces a thermal shock that is responsible for immediate cooling of grapes and must. The rapid lowering of the temperature and reduction of the environment due to the CO_2_ gaseous state can contribute to protecting phenolics from the activity of oxidase enzymes and oxygen. Moreover, the addition of dry ice to the grapes causes freezing, leading to a breakdown of cells which can then more easily release pigment and other phenolic compounds [24].

**Figure 2:**
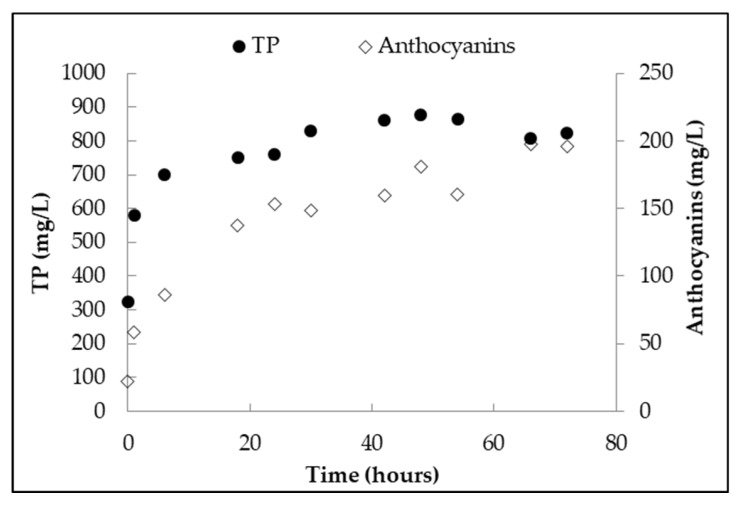
Evolution of total polyphenols (TP) and anthocyanins during processing.

The evolution of total polyphenols (TP) and anthocyanins was evaluated during processing (Figure 2). Both TP and anthocyanins were rapidly extracted from the grapes. The evolution of phenolic compounds extracted from the grapes reflected a typical solid-liquid extraction phenomenon: an almost instantaneous dissolution of “free” solutes at grape surface (i.e., leaching) was followed by diffusion of solutes from the interior of the grapes [25]. Comparing the polyphenol and anthocyanin content after 24 h with those obtained at the end of processing, about 92% of polyphenols and 78% of anthocyaninswere extracted after only 24 h. At 72 h, when the process was stopped, the concentration of anthocyanins (197 mg/L) in the juice showed a slight increase while that of TP (822 mg/L) was stable.

Phenolic composition of the juice was assayed by LC–HRMS both at the beginning (2 h) and at the end of the process (Table 1).

**Figure 3:**
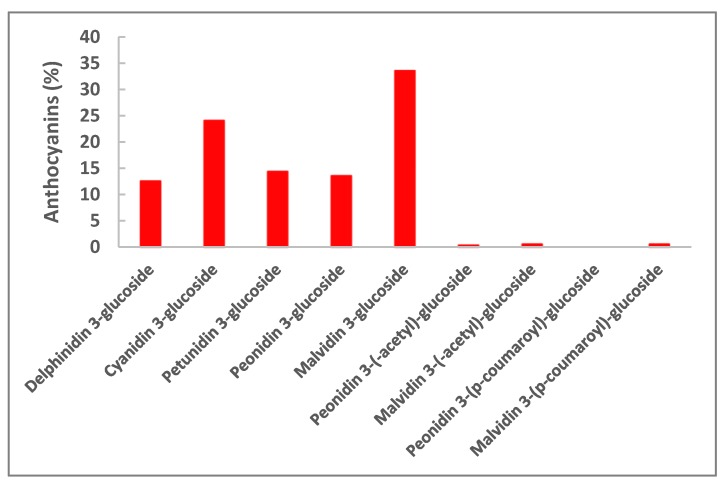
Composition (%) of anthocyanins in the juice at the end of processing.

Phenolic acids, flavonols, flava-3-ols, procyanidins and resveratrol were detected in the juice. At the beginning, the concentrations of free hydroxycinnamic acids were lower than those of their tartaric esters (caftaric, coutaric and fertaric acid), which are the most abundant forms contained in cells of grape pulp (Table 1). During processing, the concentration of the esters of hydroxycinnamic acids decreased to a significantly lower level after 72 h. The esters of hydroxycinnamic acids are rapidly oxidized via enzymatic reaction during crushing and juice extraction performed by pressing [26]. Gallic acid and flavonols were extracted in the juice during processing, reaching significantly higher concentrations by the end (72 h) with respect to those observed after 2 h. At the same time, cathechin, epichatechin, procyanidins B1 and B2, and resveratrol were efficiently extracted from the grapes. The five different anthocyanins were found in the juice in the proportions typical of Sangiovese grapes and, at lower concentration, their acetate and cumarate forms (Figure 3) [15]. The phenolic composition of powder obtained by spray-drying (S-D) with the addition of arabic gum (AG) reflected that of the juice (Table 1). Gluthatione was not detected while its oxidized form was, at a concentration of 6.6 μg/g of powder.

### 3.2. Antioxidat Activity and Effect on White Wine

The juice and powder (spray-drying (S-D) + arabic gum (AG)) were assayed for total polyphenols by Folin–Ciocalteu method and antioxidant activity (DPPH-test) (Table 2).

A phenolic concentration of 1214.6 mg CATeq/L was detected in the juice. Similar results were obtained by other authors [13], who assayed the phenolic content of juice extracted from unripe Merlot and Barbera grapes. Antioxidant activity (5345.8 µmoL TEAC/L) of the juice was almost five-fold more concentrated with respect to that observed by other authors [13]. Other compounds such as glutathione, an important constituent of grapes, can play a role in the anti-oxidant capacity of the juice [27].

About 2.6 g of juice (16.87 Brix) was required to produce 1 g of powder; 1 g of powder was composed of dry residue from juice (44.5%) and gum arabic (55.5%). Spray-dried sample had a total phenolic concentration of 2.3 mg CATeq/g of powder and antioxidant activity of 24.4 TEAC/g of powder. Regarding the total phenolic content, retention percentage for the spray-dried sample was about 70%, when compared with the juice.

The powder (S-D + AG) was used to enhance the antioxidant capacity of white wine. For this purpose, three white wines (Viognier, Chardonnay and Bellone) were combined with the powder, and their predisposition to browning was evaluated by POM-test, based on the absorbance increase at a wavelength of 420 nm after strong oxidation of the samples. The Viognier, Chardonnay and Bellone were unfinished wines with low sulfur dioxide concentrations and different chemical characteristics (Table 3).

The wines had a pH ranging from 2.90 (Bellone) to 3.35 (Viognier). Bellone was the wine with the highest acid concentration (7.1 g/L Tartaric Acid Equivalents, while Viognier showed the lowest total acidity of 5.5 g/L H_2_T.

The results of the POM-test on white wines are presented in Table 4 Each sample was tested in triplicate without and with the addition of the powders at a concentration of 2 g/L.

All the wines showed an increase of absorbance ranging from 9% (Bellone) to 34% (Chardonnay) after the addition of hydrogen peroxide. The predisposition to browning with addition of the powder was lower than that observed without any addition (Table 4). Indeed, with the addition of powder, the Chardonnay, Bellone and Viognier wines showed an increase of absorbance of 5%, 0% and −15%, respectively, after the addition of hydrogen peroxide. Several factors can contribute to the oxidizability of wine, such as phenolic composition, pH and sulphur dioxide content [28]. From our data, the anti-browning capacity of the wines does not seem related to the SO_2_ content, both free and total, while this important trait of white wine could mainly be due to the phenolic concentration and composition [28]. The obtained results show that the addition of unripe grape powder to a white wine may increase its antioxidant capacity and potentially replace the protective effect of sulphur dioxide.

## 4. Conclusions

Results from this research demonstrated that it is possible to obtain a natural occurring antioxidant complex from unripe grapes. The extract had good polyphenol content and was composed by many bioactive compounds. For the first time, an extract from unripe grapes was used to prevent oxidation of white wine with promising results. Moreover, the extract from unripe grapes could find applications in food industry as functional ingredient. The processing technique used in this study can be easily implemented in larger scale for the effective production of the extract. In conclusion, this study confirm that it is possible to exploit an undervalued by-product, such as unripe grapes, as source of natural antioxidants through a simply technique.

## Figures and Tables

**Table 1:** Phenolic composition of the juice at the beginning (2 h) and at the end (72 h) of the process, and powder obtained by spray-drying (S-D) with the addition of arabic gum (AG).

Phenolic Compounds	Concentration (mg/L) of Juice *		Concentration (μg/g of Powder) *
	Time		S-D + AG
	2 h	72 h	
**Phenolic Acids**			
Caffeic acid	0.06 ± 0.01 ^a^	0.43 ± 0.16 ^b^	0.8 ± 0.0
Coumaric acid	0.03 ± 0.00	nd	0.5 ± 0.1
Ferulic acid	1.7 ± 0.4 ^b^	0.14 ± 0.00 ^a^	29.4 ± 4.7
Caftaric acid	50.0 ± 6.6 ^b^	27.5 ± 1.7 ^a^	191 ± 5
Coutaric acid	15.9 ± 1.8 ^b^	9.4 ± 0.1 ^a^	27.6 ± 4.4
Fertaric acid	16.8 ± 3.1 ^a^	30.1 ± 3.3 ^b^	291 ± 73
Gallic acid	0.05 ± 0.00 ^a^	9.4 ± 2.1 ^b^	9.5 ± 0.3
**Flavonols**			
Quercetin	0.07 ± 0.00 ^a^	0.29 ± 0.01 ^b^	1.3 ± 0.0
Quercetin 3-*O*-glucoside	0.13 ± 0.02 ^a^	19.8 ± 0.8 ^b^	11.8 ± 0.8
Quercetin 3-*O*-glucuronide	0.38 ± 0.07 ^a^	27.0 ± 1.2 ^b^	56.6 ± 3.1
Rutin	0.02 ± 0.00 ^a^	0.40 ± 0.03 ^b^	0.4 ± 0.0
Isorhamnetin	0.04 ± 0.00	nd	0.7 ± 0.0
Kaempferol	0.02 ± 0.00	nd	0.5 ± 0.0
Myricetin	nd	0.03 ± 0.00 ^b^	nd
**Flava-3-Ols**			
(−)-Epicatechin	0.24 ± 0.02 ^a^	39.5 ± 1.6 ^b^	64 ± 12
(+) Catechin	6.3 ± 0.2 ^a^	38.4 ± 1.3 ^b^	327 ± 19
**Procyanidins**			
Procyanidin B1	0.82 ± 0.11 ^a^	23.9 ± 0.9 ^b^	19.0 ± 1.5
Procyanidin B2	0.15 ± 0.00 ^a^	2.0 ± 0.1 ^b^	10.1 ± 1.0
**Stilbenes**			
Resveratrol	0.01 ± 0.00 ^a^	± 0.00 ^b^	0.2 ± 0.0

* Data are presented as mean ± Standard Deviation (SD). Data are the mean of three replications. Significant differences (*p* < 0.05) are shown by ^a,b^ letters in the same line; nd, not detected.

**Table 2:** Total polyphenols content (TP) and antioxidant capacity of the juice and powder obtained by spray-drying (S-D) with the addition of arabic gum (AG).

Sample	Total Polyphenols * (mg CATeq/L of Juice or g of Powder)	Antioxidant Capacity * (µmoL TEAC/L of Juice or g of Powder)
Juice	1214.6 ± 37.8	5345.8 ± 119.3
S-D + AG	2.3 ± 0.01	24.4 ± 0.00

* Data are presented as means ± SD. Data are the mean of three replications. TEAC: Trolox equivalents antioxidant capacity. CATeq: (+)-catechin equivalents.

**Table 3:** General analysis of three white wines.

Sample	PH	Total Acidity (g/L as Tartaric Acid Equivalents)	Free SO_2_ (mg/L)	Total SO_2_ (mg/L)
Viognier	3.35	5.5	5.0	11.0
Chardonnay	3.06	5.6	15.5	42.3
Bellone	2.90	7.1	12.8	16.6

**Table 4:** POM-test performed without and with the addition of powder (2 g/L). Percent increase (% OX_H2O2_) of the absorbance at 420 nm.

Wine	Wine	Wine + Powder
	% OX_H2O2_	% OX_H2O2_
Viognier	12	−15
Chardonnay	34	5
Bellone	9	0
